# Comment on “Prevalence, Etiology, and Risk Factors of Tinea Pedis and Tinea Unguium in Tunisia”

**DOI:** 10.1155/2018/4859514

**Published:** 2018-04-15

**Authors:** Talel Badri

**Affiliations:** Faculty of Medicine, University of Tunis El Manar, Tunis, Tunisia

I read with great interest the article by Toukabri et al. concerning “Prevalence, Etiology, and Risk Factors of Tinea Pedis and Tinea Unguium in Tunisia” [1]. The study was about 485 mycological samples collected prospectively from 392 patients seeking dermatological care for foot and/or nail mycosis in a university hospital in Tunis, Tunisia. The authors used the chi-square test to detect statistical differences between subgroups of patients.

Although the paper is interesting, showing some significant statistical differences, the authors used only univariate analysis and not multivariate analysis. Indeed, some factors found to be significantly related with tinea pedis, such as “associated fingernails onychomycosis” and “antifungal therapy,” may not be independent factors as they seem related. A multivariate analysis would have more precisely described the factors most related to the genesis of tinea pedis.

Furthermore, the authors did not calculate the odds ratio (with its confidence interval) for every studied factor. Odds ratio is, in such studies, the most appropriate tool to specify risk factors [2]. Without multivariate analysis and odds ratio, it would be difficult to clearly identify the risk factors.

An interesting result of the study was that there was no significant statistical difference in the frequency of having tinea pedis between patients who practice ritual washing and who do not (*p*=0.410) and between patients who attend communal showers and who do not (*p*=0.631). Another study from Tunisia showed similar findings in multivariate analysis [3]. However, the authors did not discuss these “original” nonsignificant results but discussed, based on the conclusions of other studies, the mechanism by which these factors may act as risk factors (a maceration which causes fungal penetration; a source of fungal contagion). It would have been interesting to have suggestions or explanations for these differences between Tunisian findings and the other ones. Furthermore, most of the cited studies were not designed to screen risk factors and/or suffered from methodological concerns.

Prospective, multicentre, controlled studies, with multivariate analysis, would better indicate risk factors for tinea pedis and tinea unguium.
